# Apolipoprotein A-V Is a Novel Diagnostic and Prognostic Predictor in Pediatric Patients with Sepsis: A Prospective Pilot Study in PICU

**DOI:** 10.1155/2020/8052954

**Published:** 2020-01-13

**Authors:** Chunxia Wang, Yun Cui, Huijie Miao, Xi Xiong, Jiaying Dou, Lujing Shao, Xiaomeng Tang, Yucai Zhang

**Affiliations:** ^1^Department of Critical Care Medicine, Shanghai Children's Hospital, Shanghai Jiao Tong University, Shanghai 200062, China; ^2^Institute of Pediatric Critical Care, Shanghai Jiao Tong University, Shanghai 200062, China

## Abstract

**Background:**

Sepsis induces the release of lipid mediators, which control both lipid metabolism and inflammation. However, the role of serum apolipoprotein A-V (ApoA5) in sepsis is poorly understood in pediatric patients.

**Methods:**

ApoA5 was screened from serum proteomics profile in lipopolysaccharide- (LPS-) treated mice for 2 h, 24 h, and controls. Then, we conducted a prospective pilot study, and patients with sepsis admitted to a pediatric intensive care unit (PICU) were enrolled from January 2018 to December 2018. Serum ApoA5 levels on PICU admission were determined using enzyme-linked immunosorbent assays (ELISA). Blood samples from 30 healthy children were used as control. The correlation of ApoA5 with the clinical and laboratory parameters was analyzed. Logistic regression analyses and receiver operating characteristic curve (ROC) analysis were used to investigate the potential role of serum ApoA5 as a prognostic predictor for PICU mortality in pediatric patients with sepsis.

**Results:**

A total of 101 patients with sepsis were enrolled in this study. The PICU mortality rate was 10.9% (11/101). Serum ApoA5 levels on PICU admission were significantly lower in nonsurvivors with sepsis compared with survivors (*P* = 0.009). In subgroup analysis, serum levels of ApoA5 were significantly correlated with sepsis-associated multiple organ dysfunction syndrome (MODS) (*P* < 0.001), shock (*P* = 0.002), acute kidney injury (AKI) (*P* < 0.001), acute liver injury (ALI) (*P* = 0.002), and gastrointestinal (GI) dysfunction (*P* = 0.012), but not respiratory failure, brain injury, and pathogenic species (all *P* > 0.05). Correlation analyses revealed significant correlations of serum ApoA5 with Ca^2+^ concentration. Remarkably, the area under ROC curve (AUC) for serum ApoA5 levels on PICU admission was 0.789 for prediction of PICU mortality with a sensitivity of 75% and a specificity of 84.5% at a threshold value of 822 ng/mL.

**Conclusions:**

Serum ApoA5 level is associated with sepsis-associated shock, AKI, ALI, GI dysfunction, or MODS in children. Moreover, the findings of the present study suggest a prognostic value of ApoA5 in children with sepsis, and lower serum ApoA5 than 822 ng/mL predicts worse outcome in pediatric sepsis.

## 1. Introduction

Sepsis is a systemic, deleterious host response to infection leading to organ dysfunction and septic shock [1], which is common and associated with high morbidity and mortality rates in a pediatric intensive care unit (PICU). A latest report indicated that the pooled case fatality rates were higher in developing countries (31.7%) than in developed countries (19.3%) [2]. An estimated incidence of sepsis in children in China was 181/100,000 with 80% under 5 years old, and the overall case fatality rate for severe sepsis or septic shock was 34.6% [3]. Despite considerable medical advances, it is a challenge for pediatric clinicians for early assessing the progress and outcome of sepsis after admission to the PICU.

The systemic inflammatory response present in sepsis is accompanied by severe metabolic alterations with a massive release of catecholamines, stress hormones, and inflammatory mediators [4]. Impaired metabolic regulation in liver enhances mortality upon bacterial infection [5], and coordinated epigenetic and metabolic perturbations orchestrate this shift from hyper- to hypoinflammation in innate immune cells during sepsis [6]. Up to date, metabolic resuscitation or metabolic interventions might reveal to be a novel cornerstone for improving sepsis outcomes [7]. As an early clinic feature, whether biomarkers for metabolic stress might reflect the progression or outcome of sepsis is lacking evidence.

ApoA5 gene encoding apolipoprotein A-V appears to be a modulator of triglyceride homeostasis in rodents as well as in humans. Nucleotide polymorphisms in the ApoA5 gene have been associated with differences in plasma triglyceride levels in patients with familial combined hyperlipidemia [8]. The acute-phase responses in sepsis induce dyslipidemia with increased plasma triglyceride (TG) and decreased high-density lipoprotein cholesterol (HDL-C) levels [9]. A recent report in adult patients with sepsis indicated that low ApoA5 levels are associated with higher mortality, but the association became nonsignificant after adjusting for HDL-C levels [10]. However, little is known about the role of ApoA5 in pediatric patients with sepsis. Therefore, the aim of this study was to evaluate whether serum ApoA5 levels on PICU admission were correlated with the prognosis in children with a clinical diagnosis of sepsis/septic shock.

## 2. Materials and Methods

### 2.1. Analysis of Serum Proteomics of Mice Treated with Lipopolysaccharide (LPS)

Previously, we performed isobaric tags for relative and absolute quantification (iTRAQ) labeling coupled with two-dimensional liquid chromatography-tandem mass spectrometry (2D LC-MS/MS), comparing lipopolysaccharide- (LPS-) treated mice for 2 h, 24 h, and controls. The mass spectrometry proteomics data have been deposited to the ProteomeXchange Consortium *via* the PRIDE partner repository with the dataset identifier PXD014259. In the present study, we screened the different proteins involved in lipid metabolism among these serum proteomic profiles and performed Gene Ontology (GO) analysis.

### 2.2. Study Population

We conducted a prospective study, and patients with sepsis admitted to the PICU at Shanghai Children's Hospital were eligible from January 2018 to December 2018. The inclusion criteria include the following: (1) aged with over 28 days and less than 14 years and (2) diagnosed with sepsis within 24 h. The exclusion criteria include the following: (1) patients with advanced tumor or life expectancy less than 1 month and (2) patients with genetic and metabolic diseases. Sepsis was diagnosed based on the International Pediatric Sepsis Consensus Conference in 2005 [11]. Sepsis-associated complications including respiratory failure (PaO_2_/FIO_2_ < 300 mmHg in the absence of cyanotic heart disease or preexisting lung disease), acute kidney injury (AKI) (serum creatinine—2 times the upper limit of normal for age or 2-fold increase in baseline creatinine), brain injury (Glasgow Coma Score < 11), or liver injury (SALI) (total bilirubin ≥ 4 mg/dL or ALT 2 times the upper limit of normal for age) were defined according to the criteria of International Pediatric Sepsis Consensus Conference [11]. Sepsis-associated gastrointestinal dysfunction (GI) (absent bowel sounds) was defined by Surviving Sepsis Campaign International Guidelines in 2012 [12]. Sepsis-associated MODS was defined as more than 1 organ dysfunction secondary to sepsis. As a control population, we analyzed the residual blood samples from 30 healthy children to obtain the normal values of ApoA5 in pediatric population. The study protocol was approved by the local ethics committee and conducted in accordance with the ethical standards laid down in the Declaration of Helsinki (Ethics Committee of Children's Hospital affiliated to Shanghai Jiao Tong University) (approval number: 2018R039-F01). The informed consent was signed by the patients' relatives.

### 2.3. Patient's Treatment

Patients were treated with standard management including fluid therapy, antibiotics, vasoactive drugs, and other supportive therapies recommended by Surviving Sepsis Campaign International Guidelines in 2012 [12]. Some patients who transferred to other clinical departments could not exclude antibiotic treatment prior to PICU admission.

### 2.4. Blood Samples

Blood sample from children with sepsis was withdrawn exactly on PICU admission. The residual blood samples from healthy children were used as the control. After centrifugation, the serum was stored at -80°C. Serum ApoA5 levels were determined using enzyme-linked immunosorbent assays (ELISA) (MultiScience (LIANKE) Biotech, Co., Ltd., Hangzhou, China).

### 2.5. Observational Variables

The case report form was preestablished. The clinical and laboratory parameters were collected mainly including age, sex, body weight, heart rate, mean arterial pressure (MAP), systolic blood pressure (SBP), pediatric risk of mortality III (PRISM III), comorbidities, infection sites, pathogen, mechanical ventilator, and vasoactive agents. The laboratory indexes include platelet counts (PLT), infectious indexes (procalcitonin (PCT) and c-reaction protein (CRP)), biochemical parameters for organ functions (total bilirubin (TBIL), alanine aminotransferase (ALT), and lactic acid (Lac)), coagulation function (prothrombin time (PT), international normalized ratio (INR), and fibrinogen (Fib)), hemoglobin (Hb), and calcium ion (Ca^2+^). The outcome variables included the length of PICU stay and PICU survival status. The laboratory indexes were collected from the first test within 24 hours after PICU admission. For patients who had multiple admissions to PICU, only the first PICU admission was included for analysis.

### 2.6. Statistical Analysis

Data analyses were performed using STATA 15.0 MP (College Station, Texas, USA). Continuous variables were presented as the mean ± standard deviation (SD) for normal distribution data or median (Interquartile range (IQR)) for abnormal distribution data, and Student's *t*-test and the Mann–Whitney *U* test were used to compare the data, respectively. The chi-square test was used to compare the categorical data. Correlation analyses about serum ApoA5 levels and other laboratory indexes were assessed by Spearman's rank correlation due to nonnormality distributions. Odds ratios (ORs) were estimated by logistic regression models with inclusion of covariate terms chosen based on the biological plausibility of outcome. In order to appreciate the accuracy of independent predictors of PICU mortality, a ROC curve was generated. A value of *P* < 0.05 was considered statistically significant.

## 3. Results

### 3.1. Serum ApoA5 Levels Significantly Increased in Mice Treated with LPS for 24 H

**Figure 1:**
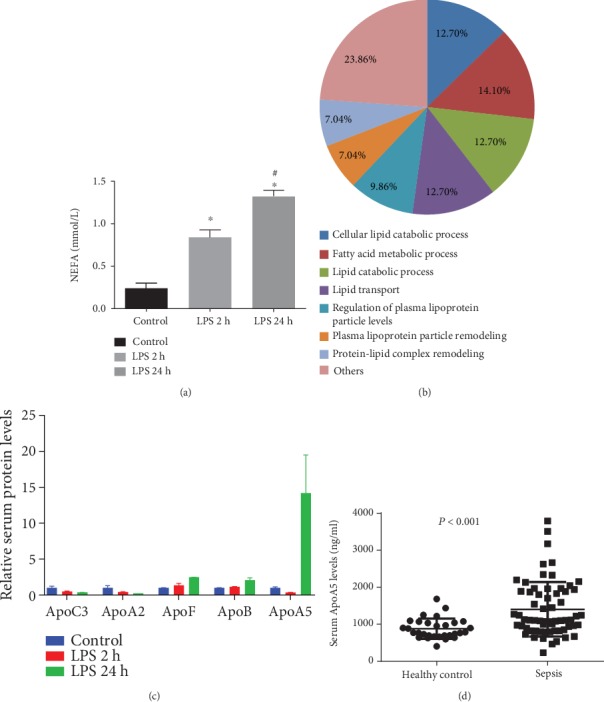
Analysis of serum proteomics of mice treated with lipopolysaccharide (LPS). (a) Serum NEFA levels. (b) Proteins involved in lipid metabolism. (c) Relative apolipoprotein (Apo) levels in serum of mice treated with LPS. (d) Serum ApoA5 levels in healthy children and pediatric patients with sepsis.

The serum nonesterified fatty acid (NEFA) levels were continuously increased in mice treated with LPS for 2 h and 24 h (Figure 1(a)). According to the GO analysis, the changed proteins involved in lipid metabolism were highly related to a fatty acid metabolic process (14.1%), cellular lipid catabolic process (12.7%), lipid catabolic process (12.7%), lipid transport (12.7%), regulation of plasma lipoprotein particle levels (9.86%), and others (Figure 1(b)). A series of apolipoproteins (Apo) including ApoC3, ApoA2, ApoF, ApoB, and ApoA5 were further quantitatively analyzed. The results showed that ApoA5 was the most significantly increased apolipoprotein at 24 h after LPS treatment (Figure 1(c)). Furthermore, serum ApoA5 levels on PICU admission in pediatric population with sepsis were significantly higher than those in the healthy children (Figure 1(d)).

### 3.2. Baseline Characteristics

To further investigate the role of ApoA5 in pediatric patients with sepsis, there were 101 pediatric patients with sepsis enrolled with a median age of 19 (5, 60) months. The baseline characteristics of patients are shown in Table 1. There were 11 cases of death with a mortality rate of 10.9% (11/101). There were significant differences between survivors and nonsurvivors in aspects of the PRISM III score (13 [5-21] *vs.* 6 [2-10], *P* = 0.007), rate of respiratory failure, shock, acute liver injury (ALI), mechanical ventilator (100% *vs.* 46.7%, *P* < 0.001), and vasoactive agents (100% *vs.* 47.8%, *P* = 0.001) (Table 1). There was no difference in terms of age, body weight, gender, infectious site, and pathogen species (all *P* > 0.05). The length of PICU stay has the tendency to be longer in nonsurvivors than in survivors but without statistical significance (*P* = 0.711) (Table 1).

### 3.3. Serum ApoA5 Levels Are Significantly Lower in Nonsurvivors than Survivors

**Figure 2:**
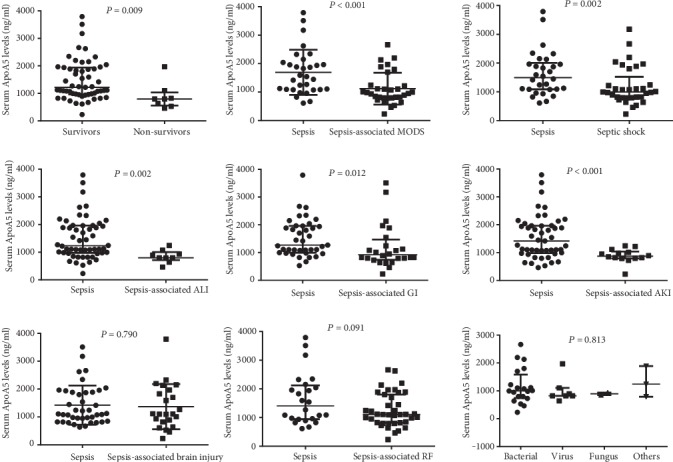
Comparison of serum ApoA5 levels in pediatric patients with sepsis complicated by different organ dysfunctions or with different pathogens.

Serum ApoA5 levels were determined at the time point of PICU admission. The results showed that ApoA5 serum concentrations and the levels of platelet count at PICU admission were significantly lower in nonsurvivors than in survivors (793.7 [583.4-962.5] ng/mL *vs.* 1219.4 [957.7-1938.6] ng/mL, *P* = 0.009; 172 [146-302] ng/mL *vs.* 292.5 [219-392] ng/mL, *P* = 0.047, respectively; Table 2). In addition, serum lactate levels at PICU admission displayed higher tendency but without statistical significance (*P* = 0.367, Table 2). To investigate the association of serum ApoA5 levels with sepsis-associated organ dysfunction precisely, we performed subgroup analysis among patients with sepsis. The serum levels of ApoA5 were decreased in patients with MODS (*P* < 0.001), shock (*P* = 0.002), AKI (*P* < 0.001), ALI (*P* = 0.002), or gastrointestinal (GI) dysfunction (*P* = 0.012), but not in patients with respiratory failure (RF) (*P* = 0.091), brain injury (*P* = 0.790) (Figure 2). Moreover, serum ApoA5 levels were not significantly different among patients with different pathogen species (*P* = 0.813) (Figure 2).

Additionally, univariate logistic regression analysis indicated that a low ApoA5 level was an independent risk factor for PICU mortality in pediatric patients with sepsis (OR: 0.998, 95% confidence interval (CI): 0.996-0.999, *P* = 0.046) (Table 3). However, there was no significant association between PLT count or TBIL and PICU mortality (both *P* > 0.05) (Table 3).

### 3.4. Serum ApoA5 Concentration Is a Diagnostic Biomarker for Sepsis and a Prognostic Predictor for PICU Mortality in Pediatric Patients with Sepsis

**Figure 3:**
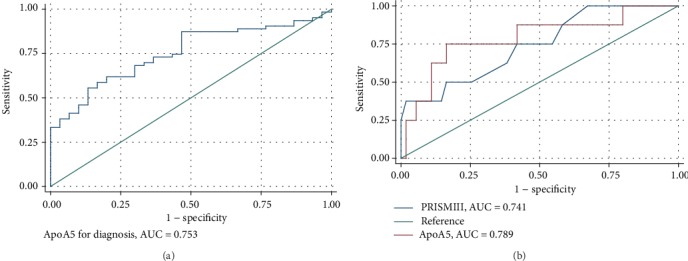
ROC analysis of serum ApoA5 concentration as a diagnostic biomarker for pediatric sepsis (a) and a prognostic biomarker for PICU mortality (b) in children with sepsis.

When we applied ROC analyses to assess the diagnostic utility of ApoA5, the serum ApoA5 level achieved an area under the ROC (AUC) of 0.753 with a 95% CI of 0.654-0.852 (Figure 3(a)). The threshold value for serum ApoA5 was 1096 ng/mL with a sensitivity of 55.56% and a specificity of 86.67% (Figure 3(a)). To evaluate the prognostic value of ApoA5 levels in pediatric patients with sepsis, ApoA5 serum levels achieved an area under the ROC (AUC) of 0.789 with a 95% CI of 0.593-0.984 (Figure 3(b)). Serum ApoA5 levels displayed the prognostic accuracy for PICU mortality with a sensitivity of 75% and a specificity of 83.6% with a cutoff value of 822 ng/mL (Figure 3(b)). In addition, an AUC for the PRISM III score to predict PICU mortality was 0.741 (95% CI: 0.552-0.930). At the cutoff value of the PRISM III score of 13, the sensitivity was 54.6% and the specificity was 85.6%.

### 3.5. Levels of Circulating ApoA5 Are Closely Correlated to Serum Ca^2+^ Levels

**Figure 4:**
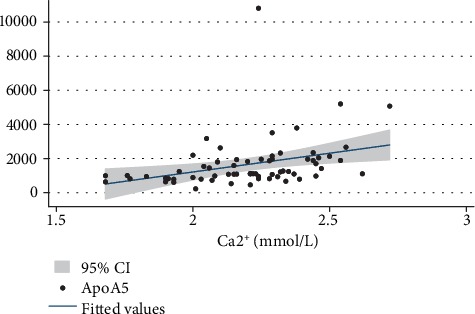
Spearman's rank correlation analysis of serum ApoA5 levels with Ca^2+^ levels in pediatric patients with sepsis.

Given the potential role of ApoA5 in liver, kidney, and GI functions in patients with sepsis, we tried to evaluate the correlation of ApoA5 levels with the indicators for organ function. Spearman's rank correlation analysis indicated that serum ApoA5 levels were found to be positively correlated with Ca^2+^ levels at the time of PICU admission (Table 4 and Figure 4), but there were no correlations with other indicators for liver, kidney, and gastrointestinal functions (data not shown).

## 4. Discussion

There is a lack of evidence to develop effective biomarkers representing lipid metabolism for assessing the progress or outcome of sepsis. In the present study, our findings show that serum ApoA5 levels in nonsurvivors were significantly lower than those in survivors with sepsis. Furthermore, the serum ApoA5 level was significantly associated with septic shock, sepsis-associated liver injury, sepsis-associated AKI, and sepsis-associated GI dysfunction.

Patients with sepsis often have a constellation of symptoms due to lipid metabolic disorder. Proteomic analysis indicated that changes in apolipoproteins and cholesterol were confirmed in the plasma, which is a possible target for future interventions of sepsis [13]. A previous study reported that pediatric patients admitted to the PICU with severe sepsis or septic shock displayed lower levels of cholesterol and high-density lipoprotein (HDL) and low-density lipoprotein (LDL) cholesterol concentrations accompanied by reductions in apolipoproteins levels, which suggested that these apolipoproteins could be used as potential biomarkers for sepsis [14]. In the present study, we firstly screened ApoA5 based on serum proteomics of mice treated with LPS. Serum ApoA5 levels increased after LPS administration for 24 h, which was the most significantly changed apolipoprotein in serum. Consistently, hepatic ApoA5 mRNA expression was upregulated at 8 to 24 hours after endotoxin injection in mice, and ApoA5 protein was increased at 16 hours [15]. In our present study, elevated serum ApoA5 levels were confirmed in pediatric patients with sepsis, which were significantly higher when compared with healthy children. Importantly, an AUC of serum ApoA5 for discriminating sepsis from control was 0.753 with a 95% CI of 0.654-0.852. All these results suggested that ApoA5 as an acute-phase protein responding to infection could be an additional biomarker for sepsis.

Serum ApoA5 levels were significantly low in nonsurvivors with sepsis. Consistently, a recent report in adult patients with sepsis indicated that low ApoA5 levels are associated with higher mortality [10]. Compared with the report by Ngaosuwan et al. [10], the absolute value of serum ApoA5 was significantly higher in pediatric patients with sepsis than in adult patients with sepsis. Though we confirmed that serum levels of ApoA5 were not associated with age and gender in our present study, the difference in serum ApoA5 levels between children and adults needs to be investigated further. In addition, serum ApoA5 levels were not significantly different among patients who are infected with bacteria, virus, fungi, and others. It is consistent with the results from the adult patients who had gram-positive sepsis and those who had gram-negative sepsis [10]. Given the complexity of infection in the PICU, the stability of serum ApoA5 levels suggested that ApoA5 might be a general biomarker for adaptive response regardless of source of infection during sepsis. According to the result of ROC analysis, serum ApoA5 levels achieved an AUC of 0.789 (95% CI: 0.593-0.984) as a prognostic biomarker for PICU mortality with a sensitivity of 75% and a specificity of 83.6% at a cutoff value of 822 ng/mL. In comparison, the PRISM III score, as a well-known prognostic indicator, got a relatively lower value of an AUC of 0.741 (95% CI: 0.552-0.930) and a relatively lower sensitivity was 54.6% at the cutoff value of a PRISM III score of 13 than that of serum ApoA5. To the best of our knowledge, this is the first report about serum ApoA5 as a prognostic biomarker of pediatric sepsis. All these results need further clinical researches in a large population with pediatric sepsis.

ApoA5 is a well-known apolipoprotein involved in lipid metabolism. Deficiency of ApoA5 results in hypertriglyceridemia, whereas overexpression of ApoA5 leads to a reduction in triglyceride levels [16]. Furthermore, the relationship between ApoA5 and triglyceride levels may depend on the underlying genetic determinants, race, and status of the patients [17–20]. In our study, serum ApoA5 levels could not be affected by age, gender, body weight, and pathogens, and it was specifically related the liver, kidney, and GI dysfunctions. Given the important roles of the liver, kidney, or GI in lipid metabolism, serum ApoA5 levels mainly might be related to the metabolic stress during sepsis. Moreover, it is surprising that serum ApoA5 levels were significantly related to Ca^2+^ concentration. During sepsis, erythrocyte with increased cytosolic Ca^2+^ activity is characterized by lipid scrambling of the cell membrane leading to phosphatidylserine exposure at the erythrocyte surface, then adheres to vascular walls or may be engulfed by macrophages equipped with phosphatidylserine receptors, named eryptosis [21]. Here, we are not sure about the potential mechanism of the relationship between ApoA5 and Ca^2+^ concentration, which needs to be verified in the future.

There were several limitations in this study. This is a single-center, small sample size, prospective pilot study. The time course of changes in ApoA5 levels after PICU admission was not characterized due to the lack of blood sample. Because of the lack of a laboratory test about HDL or triglyceride, the correlation between ApoA5 and HDL or triglyceride was absent. Nevertheless, to the best of our knowledge, this is the first report about the serum ApoA5 level as a stable and powerful prognostic predictor in pediatric patients with sepsis.

## 5. Conclusions

The serum ApoA5 level is associated with indicators of illness severity like shock, AKI, ALI, GI dysfunction, or MODS in pediatric patients with sepsis. Moreover, the findings of the present study suggest a prognostic value of ApoA5 in children with sepsis, and further studies are needed to confirm this conclusion in a well-designed prospective study with a large population.

## Figures and Tables

**Table 1:** Baseline characteristics of patients with sepsis.

	Total (*n* = 101)	Survivor (*n* = 90)	Nonsurvivor (*n* = 11)	*P*
Age (month)	19 (5-60)	19 (7-62)	5 (2.5-45)	0.357
Body weight (kg)	12 (7.5-18.75)	12 (8-19)	7.5 (6-15.5)	0.217
Gender (male, %)	58	54	4	0.134
From emergency	71	66	5	0.056
Respiratory rate (/min)	36 (30-46)	35 (28-45)	40.5 (38-60)	0.101
Heart rate (beats/min)	150 (121-179)	150 (120-175)	160 (150-198)	0.100
MAP (mmHg)	86 (75.7-97.3)	86 (74.6-96.7)	92 (80-107)	0.208
SBP (mmHg)	101 (88-112)	100.5 (88-112)	102 (92-115)	0.563
PRISM III	6 (2-12)	6 (2-10)	13 (5-21)	0.007
Co-morbidities				
Respiratory failure, *n* (%)	60 (59.4)	49 (54.4)	11 (100)	0.004
Shock, *n* (%)	45 (44.6)	35 (38.9)	10 (90.9)	0.001
Gastrointestinal disorder, *n* (%)	35 (34.7)	29 (32.2)	6 (54.5)	0.142
Liver injury, *n* (%)	11 (10.9)	7 (7.8)	4 (36.4)	0.004
Acute kidney injury, *n* (%)	17 (16.8)	15 (16.7)	2 (18.2)	0.899
Infection site				0.491
Respiratory system, *n*	52	45	7	
Gastrointestinal system, *n*	24	23	1	
Urine system, *n*	1	1	0	
Skin and soft tissue, *n*	1	1	0	
Central nervous system, *n*	14	11	3	
^a^Others, *n*	9	9	0	
Pathogen				0.887
Bacterial, *n*	57	51	6	
Virus, *n*	29	25	4	
Fungi, *n*	2	2	0	
^b^Others, *n*	13	12	1	
Mechanical ventilator, *n* (%)	53 (52.5)	42 (46.7)	11 (100)	< 0.001
Vasoactive agents, *n* (%)	54 (53.7)	43 (47.8)	11 (100)	0.001
Length of PICU stay (day)	13 (8-21)	13 (8-20)	17 (4-29)	0.711

MAP: mean arterial pressure; SBP: systolic blood pressure; PRISM III: pediatric risk of mortality III; ^a^Others: more than one infection site found in the patient; ^b^Others: mixed infection, more than one pathogen species found in the patient.

**Table 2:** The laboratory indexes of patients with sepsis.

	Total (*n* = 101)	Survivor (*n* = 90)	Nonsurvivor (*n* = 11)	*P*
ApoA5 (ng/mL)	1107.7 (849.4-1889.5)	1219.4 (957.7-1938.6)	793.7 (583.4-962.5)	0.009
Male	1231.2 (821.8-2042.8)	1566.9 (1006.7-2132.6)	793.7 (627.2-807.8)	0.013
Female	1104.3 (913.5-1693.9)	1106.6 (941.7-1693.9)	870.0 (583.4-1536.1)	0.278
PCT	1.22 (0.15-7.9)	1.19 (0.13-7.9)	1.82 (0.15-10.13)	0.831
Ca^2+^ (mmol/L)	2.24 (2.08-2.35)	2.24 (2.08-2.36)	2.21 (2.03-2.29)	0.557
CRP (mg/L)	21 (5-81)	20.5 (5-81)	23 (5-150)	0.638
Lac (mmol/L)	1.1 (0.7-1.9)	1.1 (0.7-1.9)	1.5 (0.9-2.6)	0.367
Hb (g/L)	106.7 ± 21.2	107.7 ± 20.4	98.5 ± 27.2	0.180
INR	1.13 (1.05-1.25)	1.13 (1.06-1.25)	1.11 (1.03-1.19)	0.570
PT (s)	13.1 (12.2-14.4)	13.1 (12.3-14.4)	12.6 (12-13.8)	0.483
Fib (g/L)	2.8 (1.8-4.0)	2.8 (1.8-4.2)	2.2 (0.7-2.9)	0.113
ALT (U/L)	20 (14-43)	19.5 (14-43)	26 (19-71)	0.198
TBIL (*μ*mol/L)	7.2 (4.7-13.9)	6.6 (4.7-11.6)	13.9 (8.3-17.9)	0.063
PLT (×10^9^/L)	282 (187-364)	292.5 (219-392)	172 (146-302)	0.047

PCT: procalcitonin; CRP: C-reactive protein; Lac: lactate; Hb: hemoglobin; INR: international normalized ratio; PT: prothrombin time; Fib: fibrinogen; ALT: alanine aminotransferase; TBIL: total bilirubin; PLT: platelet.

**Table 3:** Univariate logistic regression analysis about ApoA5, platelets, and total bilirubin for PICU mortality in patients with sepsis.

	Univariate logistic analysis	
	OR (95% CI)	*P*
ApoA5	0.998 (0.996-0.999)	0.046
TBIL	1.000 (0.995-1.005)	0.969
PLT	0.995 (0.990-1.000)	0.069

TBIL: total bilirubin; PLT: platelets.

**Table 4:** Spearman's rank correlation.

ApoA5	Coef.	SE	Adjusted *R*-squared	*P*
Ca^2+^	1307.1	396.9	0.137	0.002

## Data Availability

All data generated or analyzed during this study are available from the corresponding author upon reasonable request.

## Abbreviations

ApoA5: Apolipoprotein A5
AST: Aspartate transaminase
ALT: Alanine transaminase
AUC: Area under the ROC curve
Ca^2+^: Calcium ion
ELISA: Enzyme-linked immunosorbent assays
Fib: Fibrinogen
GO analysis: Gene Ontology analysis
Hb: Hemoglobin
HDL: High-density lipoprotein
IL-6: Interleukin-6
INR: International normalized ratio
iTRAQ: Isobaric tags for relative and absolute quantification
Lac: Lactic acid
LDL: Low-density lipoprotein
LPS: Lipopolysaccharide
MAP: Mean arterial pressure
PICU: Pediatric intensive care unit
PRISM III: Pediatric risk of mortality III
PT: Prothrombin time
ROC: Receiver operating characteristic curve
SBP: Systolic blood pressure
TNF-*α*: Tumor necrosis factor-*α*.

## Additional Points

*Highlights*. ApoA5 is a novel sepsis biomarker that can discriminate pediatric patients with sepsis from healthy controls. ApoA5 can predict mortality among septic children with a sensitivity of 75% and a specificity of 83.6% at a threshold value of 822 ng/mL. ApoA5 is associated with indicators of sepsis severity like septic shock, sepsis-associated multiple organ dysfunction syndromes (MODS), acute kidney injury (AKI), acute liver injury (ALI), and gastrointestinal (GI) dysfunction, but not respiratory failure, brain injury, and pathogenic species
